# Phosphorylated Chemiluminescent Probe Based on Spiro-cyclobutyl-phenoxy-dioxetane for the In Situ Light-Activated Sensing of Alkaline Phosphatase

**DOI:** 10.1021/acs.analchem.5c07061

**Published:** 2026-04-07

**Authors:** Cheng-Jun Zhao, Wen-Zhen Gui, Tian-Hao Wang, Xi-Le Hu, Hong-Yang Zhang, Tony D. James, Xiao-Peng He

**Affiliations:** † Key Laboratory for Advanced Materials and Joint International Research Laboratory of Precision Chemistry and Molecular Engineering, Feringa Nobel Prize Scientist Joint Research Center, School of Chemistry and Molecular Engineering, 47860 East China University of Science and Technology, 130 Meilong Rd., Shanghai 200237, China; ‡ The International Cooperation Laboratory on Signal Transduction, Eastern Hepatobiliary Surgery Hospital, National Center for Liver Cancer, Shanghai 200438, China; § Department of Chemistry, 1555 University of Bath, Bath BA2 7AY, U.K.; ∥ School of Chemistry and Chemical Engineering, Henan Normal University, Xinxiang 453007, China

## Abstract

Alkaline phosphatase (ALP) plays important biological roles for many living species, including bacteria. Here, we developed an ALP probe (**CL-A1**) using the dephosphorylation of an in situ light-activated chemiluminescent species based on a spiro-cyclobutane-substituted dioxetane scaffold. A sequential light-induced oxidation and ALP-mediated dephosphorylation of **CL-A1** rapidly generated intense chemiluminescence (CL), which was stronger than that of the commercial CL substrates **AMPPD** and **APS-5** tested in the presence of a signal enhancer. Analysis using *Escherichia coli* strains confirmed the applicability of **CL-A1** for the sensitive detection of endogenous ALP activity using in situ light activation.

## Graphical abstract

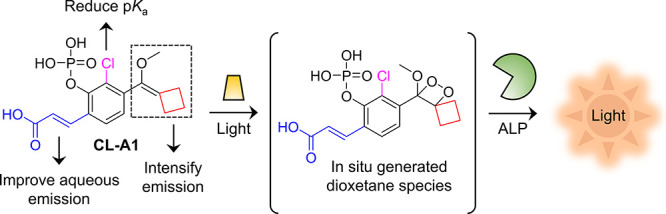

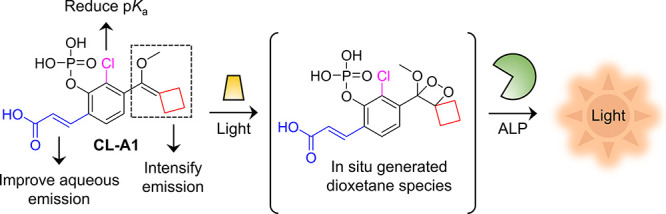

Alkaline phosphatase (ALP) is a class of enzymes widely distributed in nature that catalyzes the hydrolysis of phosphate esters. It is found in both humans and bacteria, playing essential physiological and ecological roles.^1^ ALP modulates a variety of metabolic and catabolic processes through the hydrolytic removal of phosphate groups from proteins, nucleosides, and various other substrates.^2,3^ In bacteria, ALPs are mainly encoded by the phoA, phoD and phoX gene families. Exhibiting phylogenetic divergence and ecological adaptation, ALPs can be localized in the cytoplasm, inner membrane, periplasm, outer membrane, and secreted out of cells.^4–7^ The sensitive detection of bacterial ALP could help better understand its regulatory role in bacterial survival as well as aid the diagnosis of bacterial infections. In addition, abnormal levels in ALP activity are also associated with a variety of other human diseases.^8–12^

Clinically, colorimetric substrates such as p-nitrophenyl phosphate are commonly used for ALP detection. To overcome problems associated with colorimetric analyses in terms of low sensitivity and background interference, mechanistically diverse small-molecule fluorescent probes^13–21^ have been developed. Recent research has also seen an emerging trend in the development of chemiluminescence (CL)-based probes for ALP sensing. CL assays are among the most sensitive analytical methods for the detection of enzymatic activities due to their high signal-to-noise ratio.^22–24^ CL probes typically undergo an oxidative step to form an unstable strained peroxide that rapidly decomposes to produce an emissive substance in an excited-state. Subsequently, on decay to the ground state, light is emitted.^25^

Oxidation is a common mechanism by which to activate flash-mode CL substrates such as luminol and oxalate.^26^ In contrast, spiro-adamantyl-phenoxy-1,2-dioxetanes,^27^ another widely used CL species, do not require oxidation to release luminescence. Instead, generation of their phenolate ion initiates an electron transfer to the dioxetane leading to O–O bond cleavage, and the subsequent C–C bond breakage produces an excited benzoate that can glow for minutes to hours. Significantly, Shabat and co-workers found that incorporation of an electron-withdrawing group at the ortho-position of a spiro-adamantyl-phenoxy-1,2-dioxetane substantially improved its emissive intensity in water.^28^ More recently, to induce the CL state of these species to a flash mode, the same group synthesized spiro-cyclobutyl-phenoxy-dioxetane derivatives resulting in up to 1000-fold greater emission enhancement.^29^ Using this elegant strategy, the ultrasensitive detection of enzymatic activities^30^ and reactive species^31^ can be achieved.

Here, we designed and synthesized a new CL probe (**CL-A1**) based on the dephosphorylation of spiro-cyclobutyl-phenoxy-dioxetane for the ultrasensitive detection of ALP. A sequantial in situ light-driven oxidation followed by ALP-mediated hydrolysis gave rise to a 7.5 × 10^–6^ U mL^–1^ limit of detection for ALP with outstanding selectivity over other competing enzymes and ions. Notably, the CL intensity of **CL-A1** is much stronger than that of two commercial CL-based ALP substrates, 3-(2’-spiroadamantane)-4-methoxy-4-(3’-phosphoryloxy)­phenyl-1,2-dioxetane (**AMPPD**) and sodium ((4-chlorophenyl)­thio)­(10-methylacridin-9­(10H)-ylidene)­methyl phosphate (**APS-5**).

## Experimental Section

### General

All purchased chemicals and reagents are of analytical grade unless otherwise noted. Alkaline phosphatase (ALP), α-glucosidase (α-Glc), glucose oxidase (GOD) and human serum albumin (HSA) were purchased from Sigma-Aldrich. β-Galactosidase (β-Gal) was purchased from Shanghai Yuanye Bio-Technology Co., Ltd. α-Fetoprotein (AFP) and rabbit anti-AFP were purchased from Feipeng Biotechnology Co., Ltd. Sapphire-II was purchased from Thermo Fisher Scientific (Bedford, MA, USA). ALP-conjugated goat antirabbit secondary antibody was purchased from Shanghai Yuanye Bio-Technology Co., Ltd. ^1^H NMR, ^13^C NMR and ^31^P NMR spectra were recorded on a Bruker AM 400 MHz spectrometer with tetramethylsilane (TMS) as the internal reference. High-resolution mass spectra (HRMS) were recorded with a Waters Micromass LCT mass spectrometer. LC-MS was performed on an Agilent 1290 Infinity UHPLC coupled with 6530 Q-TOF MS. Chemiluminescence was recorded on a Synergy H4 (BioTek Instruments, Inc.). LED lamp (590 nm, Shenzhen Benstartech Electronics Co., Limited) was used as excitation and the light intensity was 118 lm.

### In Situ Light-Driven Oxidation of CL-A1

To a white 96-well plate, 10 μL **CL-A1** (100 μM in deionized water) and 10 μL MB (50 μM in deionized water) were sequentially added to 70 μL deionized water. After mixing with gentle pipetting, the mixture was irradiated under yellow light (590 nm, 10 W) for 30 min. After oxidation, the as-prepared solution was immediately used for enzymatic sensing.

### Detection of Bacterial ALP

Chemiluminescent kinetic profiles were recorded using a Synergy H4 microplate reader. The injector settings were fixed with a measuring time of 600 s and an interval time of 10 s. Measurements were conducted in a white 96-well plate. Each well contained 60 μL deionized water (pH 7.0), 10 μL **CL-A1** (100 μM in water), 10 μL MB (50 μM in water) and 20 μL *Escherichia coli* (*E. coli*, ATCC25922, DH5α, TOP10 or MG1655) (1 × 10^9^ CFU mL^–1^) obtained from American Type Culture Collection (ATCC) with a final well volume of 100 μL. After mixing with gentle pipetting, the mixture was irradiated under yellow light for 30 min. Then, *E. coli* was added, and the measurement was initiated immediately. Measurements were repeated three times to ensure reproducibility. For the competition assay, (−)-*p*-bromolevamisole oxalate (***L-p*-BT**) oxalate was pretreated with ALP or *E. coli* for 30 min prior to addition of the probe.

## Results and Discussions

The CL luminophore used for the construction of the probe is based on the spiro-cyclobutyl-phenoxy-dioxetane scaffold developed by Shabat and co-workers.^29^ An electron-withdrawing group (methyl acrylate) was incorporated ortho to the phenol group to improve the aqueous emission, and an ortho chlorine group was also added to reduce the p*K*_a_ of the phenol, thus increasing the decomposition rate of the compound at physiological pH.^32^ To obtain the CL probe, the phenolic group of intermediate **1** prepared according to literature methods^29^ was phosphorylated in the presence of phosphorus trichloride and 3-hydroxypropionitrile as a protecting group to produce phosphate **2** (Scheme 1). Deprotection with sodium methoxide (1 M in methanol) afforded **CL-A1** in a two-step yield of 62.8%. A subsequent oxidation of **CL-A1** to the dioxetane derivative generated the desired, active CL species for sensing. However, considering its instability,^33^ we estabilizhed a light-driven oxidation protocol by which to in situ generate this dioxetane species with the assistance of light.

**Scheme 1:**
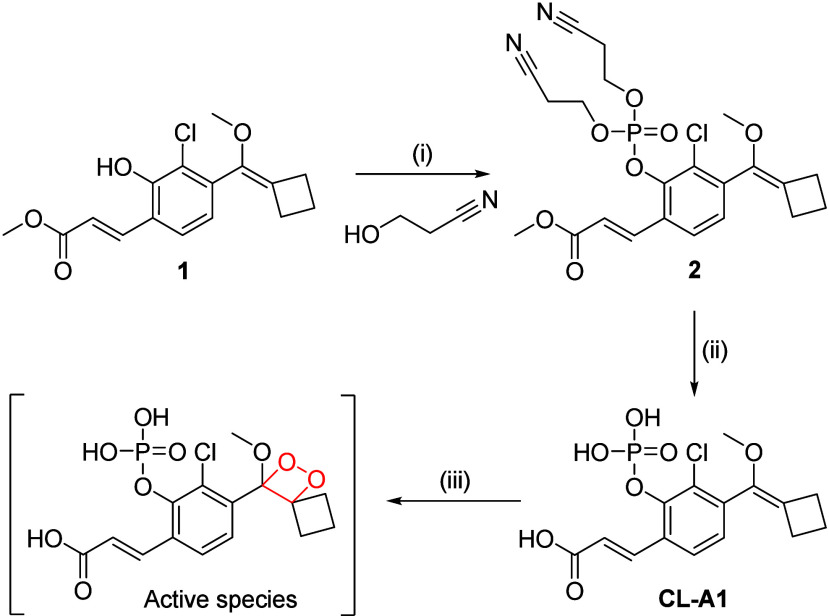
Preparation of the CL Probe^a^

a Reagents and conditions: (i) POCl_3_ in pyridine, 0 °C to room temperature (r.t.), 16 h; (ii) NaOMe in MeOH, r.t., 5 h; (iii) Methylene blue, light (λ = 590 nm) in deionized water, r.t., 30 min.

Using a microplate reader (Synergy H4), we validated this protocol by incubating the probe with methylene blue (**MB**) used as a light sensitizer in deionized water. **MB** is capable of producing ^1^O_2_ under light irradiation, and then the electron-rich enolether unit in **CL-A1** undergoes an oxidative reaction with ^1^O_2_ to produce the active intermediate that can be hydrolyzed by ALP to produce chemiluminescence (Figure S1). Light with a wavelength of 590 nm was used for irradiating the probe solution for 30 min (a photo displaying the light-driven in situ oxidation reaction is shown in Figure S2).^34^ Then, ALP (0.16 U mL^–1^) purified from bovine intestinal mucosa was immediately added to avoid decomposition of the active species. This led to sharp CL enhancement with respect to control groups in which only light or **MB** was present (Figure 1a). Notably, a ∼14,000-fold increase in CL intensity was observed for ∼30 min of incubation with ALP, suggesting a flash-mode CL burst of the probe.

**Figure 1:**
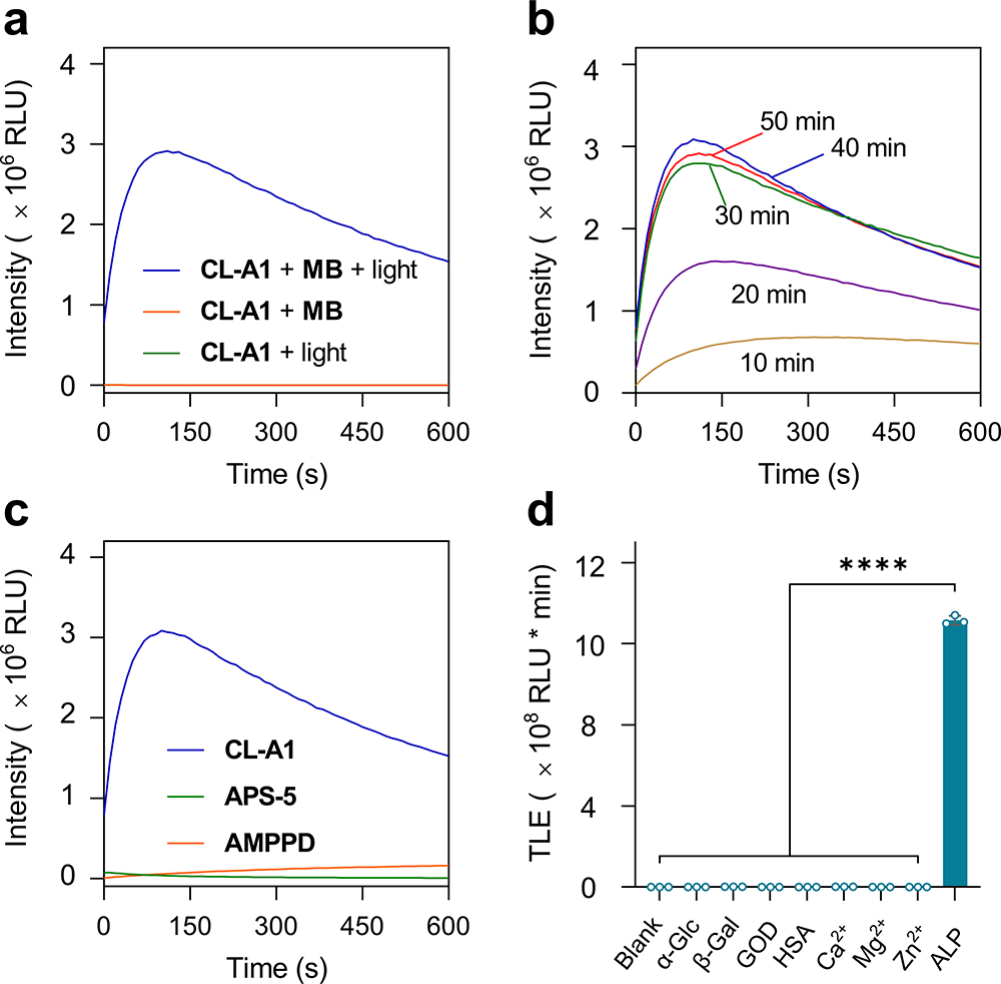
(a) Time-dependent CL generation of **CL-A1** (10 μM) in the absence and presence of **MB** (5 μM) and light with ALP (0.16 U mL^–1^) for 30 min. (b) Time-dependent CL generation of **CL-A1** (10 μM) in the presence of ALP (0.16 U mL^–1^) at different light exposure times. (c) Time-dependent CL generation of **CL-A1** (10 μM), **AMPPD** (100 μM with 10% Sapphire-II) and **APS-5** (100 μM with 10% Sapphire-II) in the presence of ALP (0.16 U mL^–1^). (d) Total light emission (TLE) of **CL-A1** (10 μM) in the presence of other analytes including α-glucosidase (α-Glc, 0.1 U mL^–1^), β-galactosidase (β-Gal, 0.1 U mL^–1^), glucose oxidase (GOD, 0.1 U mL^–1^), human serum albumin (HSA, 50 mg mL^–1^), Ca^2+^ (10 mM), Mg^2+^ (10 mM) and Zn^2+^ (10 mM). Time-dependent CL generation of **CL-A1** in deionized water on a Synergy H4 microplate reader; time-dependent CL generation of **AMPPD** and **APS-5** in Tris-HCl (50 mM, pH 9.0) on a Synergy H4 microplate reader. ****P < 0.0001 (*n* = 3).

A time-dependent analysis was then carried out, and the result showed that exposure of **CL-A1** to light for 30 min in the presence of **MB** reached equilibrium for ALP detection (Figure 1b). This suggests that **CL-A1** reacted to generate the dioxetane derivative in 30 min. We also found that the presence of 5 μM **MB** was enough for completion of the reaction (Figure S3), and time-dependent analysis by Liquid Chromatograph Mass Spectrometer (LC-MS) confirmed the complete conversion of **CL-A1** to the active species in 30 min (Figure S4). In addition, we found that the CL response of the probe remained stable from pH 7.0–9.0. However, the intensity decreased when the pH was 10.0, and was almost quenched at pH 11.0 (Figure S5). As a consequence, the following biosensing studies were carried out at pH 7.0, which is closer to physiological pH. We also tested the photostability of the probe, and the results showed that its CL intensity maintained stable even after 6 h of white light irradiation (Figure S6). We also prepared a classic adamantyl-dioxetane probe **CL-P1** as control for direct comparison of sensitivity (Scheme S1). Under the same analytical conditions, we found that **CL-A1** was much more sensitive than **CL-P1** even at a 10-fold lower concentration (Figure S7), confirming the success of our probe design strategy.

We also used two commercial CL-based ALP substrates, **AMPPD** and **APS-5**, for comparison under the optimal analytical conditions. We determined a stronger CL generation (at their respective emission maximum) for **CL-A1** than that of **AMPPD** and **APS-5** in the presence of a commercial signal enhancer (Sapphire-II) (Figure 1c). Subsequently, the selectivity of **CL-A1** for ALP was evaluated. A range of biospecies including α-glucosidase (α-Glc), β-galactosidase (β-Gal), glucose oxidase (GOD), human serum albumin (HSA), Ca^2+^, Mg^2+^ and Zn^2+^ were incubated with the probe under identical conditions. CL responses were recorded on the microplate reader. As shown in Figure 1d, negligible emission enhancements were observed for the other enzymes, underscoring the outstanding selectivity of **CL-A1** for ALP.

Next, the concentration-dependent CL response of **CL-A1** to ALP was determined. We observed a monotonic enhancement in CL intensity for the probe with increasing ALP concentrations from 0.16–5 × 10^–5^ U mL^–1^ (Figure 2a). A linear calibration curve was obtained over this concentration range, and a limit of detection (LOD) of 7.5 × 10^–6^ U mL^–1^ was determined (3*σ*/*k*, where *σ* is the standard deviation by repeated measurements of the blank and *k* is the slope of the curve). A systematic comparison suggests 1–3 orders of magnitude lower LOD for **CL-A1** than previously developed fluorescence-based and CL-based ALP probes (Table S1).^13,14,19–21,35–39^

**Figure 2:**
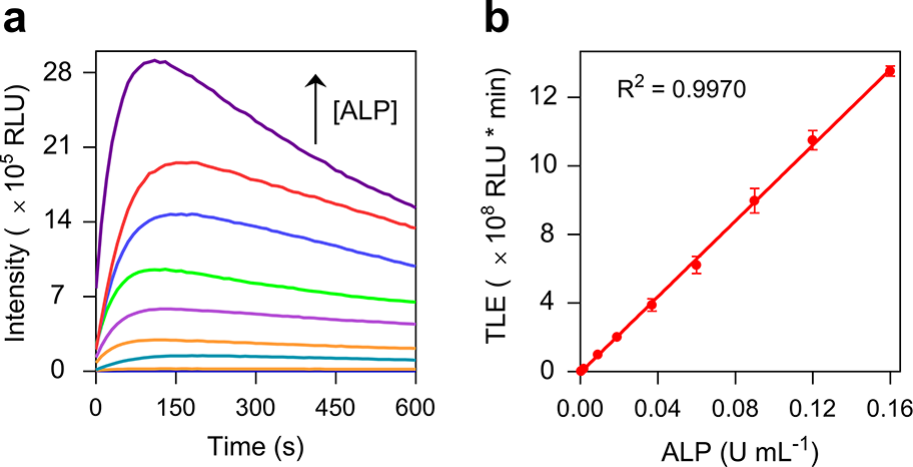
(a) Time-dependent CL generation of **CL-A1** (10 μM) with increasing concentrations of ALP (from bottom to top curve: 5.0 × 10^–5^, 2.0 × 10^–4^, 4.2 × 10^–4^, 1.7 × 10^–3^, 9 × 10^–3^, 1.9 × 10^–2^, 3.7 × 10^–2^, 6.0 × 10^–2^, 0.09, 0.12, and 0.16 U mL^–1^). (b) Plotting the TLE of **CL-A1** (10 μM) as a function of ALP concentration. All measurements were performed in H_2_O on a Synergy H4 microplate reader.

### Applicability of CL-A1 for Sensing Bacterial ALP

*E. coli*, which is known to express ALP, was used.^40^ The expression of ALP in *E. coli* is primarily regulated by the phoA gene.^41^ Four *E. coli* strains including ATCC25922, DH5α, TOP10 and MG1655 used were subjected to measurement by real-time quantitative polymerase chain reaction (RT-qPCR). The result indicated a relatively higher phoA gene in ATCC 25922 than in other strains (Figure 3a). Interestingly, a subsequent detection of these four *E. coli* strains with **CL-A1** led to the observation of a similarly higher CL intensity for ATCC 25922 than for other strains (Figure 3b). This indicates the reliability of the probe for sensing endogenous ALP. We also found consistently higher CL intensities in the lysate of these strains than in their intact forms (Figure 3c). This suggests that the ALP molecules are more thoroughly released in the lysates facilitating sensing.

**Figure 3:**
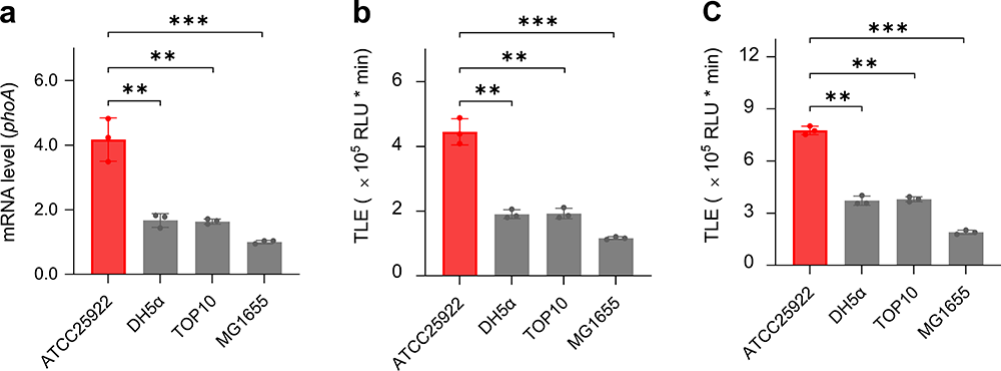
(a) mRNA level of an ALP-encoding gene (phoA) in 4 types of *E. coli* (ATCC25922, DH5α, TOP10 and MG1655) measured by RT-qPCR. TLE of **CL-A1** (10 μM) with 4 types of *E. coli* (ATCC25922, DH5α, TOP10 and MG1655) in their (b) intact and (c) lysed form. **P < 0.01, **P < 0.001 (*n* = 3).

Next, a competition assay was performed to validate ALP-dependent sensing using (−)-*p*-bromolevamisole oxalate (***L-p*-BT**), a known ALP inhibitor.^42^ We first observed that the presence of the inhibitor (1 mM) suppressed the emission of **CL-A1** with purified ALP (Figure 4a). We also found that preincubation of the inhibitor with all bacteria in their intact (Figure 4b) and lysed form (Figure 4c) led to decrease in CL intensity of the probe. This confirms the specificity of the probe for ALP sensing as well as its potential to be used for the screening of ALP inhibitors.

**Figure 4:**
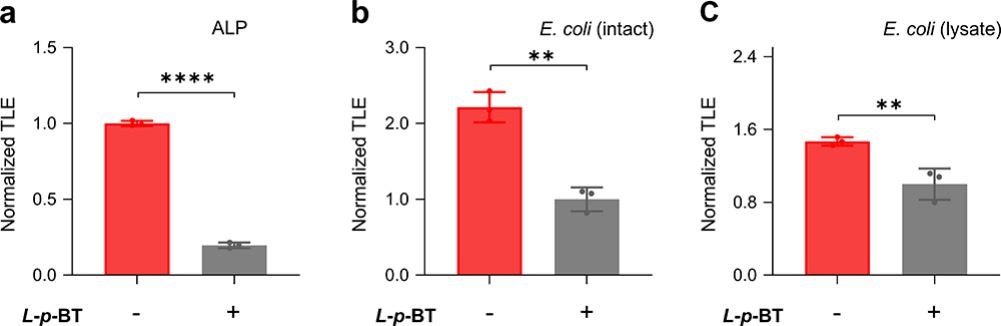
(a) Normalized TLE of **CL-A1** (100 μM) in the absence and presence of ALP (0.16 U mL^–1^) and ***L-p*-BT** (1 mM). Normalized TLE of **CL-A1** (10 μM) in the absence and presence of *E. coli* (ATCC25922) (2.0 × 10^8^ CFU mL^–1^) and ***L-p*-BT** (1 mM) in their (b) intact and (c) lysed form. All measurements were performed in H_2_O on a Synergy H4 microplate reader. ****P < 0.0001, **P < 0.01 (*n* = 3).

Finally, the feasibility of **CL-A1** for immunoassays was examined by measuring α-fetoprotein (AFP, a primary liver cancer biomarker) concentrations in a serum-mimicking solution containing biological species commonly found in the human blood. Anti-AFP-antibody-modified magnetic beads were used as the capture reagent, an anti-AFP antibody was used as primary antibody, an ALP-modified IgG was used as secondary antibody, and **CL-A1** was used as the CL substrate to establish the immunoassay system. Different concentrations of AFP were added to the prepared solutions with a starting concentration of 10 × 10^–4^ g mL^–1^, and then the final concentrations (total of the starting and added AFP concentrations) were quantified using the system (Table S2). Recovery rates ranging from 97.6–102.9% were determined for the CL immunoassay with relative standard deviations (RSD) of ≤ 5.1%. These results demonstrate the reliability of **CL-A1** as a substrate for immunoassays.

## Conclusions

In summary, a chemiluminescent probe **CL-A1** for the ultrasensitive detection of ALP was developed based on a sequential in situ light-driven oxidation and dephosphorylation. The probe was shown to be much more sensitive than two commercial CL-based ALP substrates. **CL-A1** was also applicable for the sensing of bacterial ALP with the results agreeing with those obtained from PCR. This study offers a potent chemical tool for the evaluation of ALP biology. In the meantime, the probe also exhibits the potential to be exploited as an ALP substrate for chemiluminescent immunoassays facilitating in vitro diagnosis.^43^

## Supplementary Material



## Notes

Additional experimental procedures and S-figures supporting the experimental results discussed in the main text, ^1^H NMR, ^13^C NMR and ^31^P NMR spectra for all compounds, and additional references (PDF)
