# Supplementary material for: Phosphorylated Chemiluminescent Probe Based on Spiro-cyclobutyl-phenoxy-dioxetane for the In Situ Light-Activated Sensing of Alkaline Phosphatase
Source: Anal Chem. 2026 Apr 7;98(15):11064–9. doi: 10.1021/acs.analchem.5c07061 (PMC13103926; doi:10.1021/acs.analchem.5c07061)
Supplement: Supplementary file 1 [file ac5c07061_si_001.pdf]

Supporting Information

Phosphorylated chemiluminescent probe based on spiro-cyclobutyl-phenoxy-dioxetane for the in-situ light-activated sensing of alkaline phosphatase

Cheng-Jun Zhao,[1] Wen-Zhen Gui,[1] Tian-Hao Wang,[1] Xi-Le Hu,[1]* Hong-Yang Zhang,[1]* Tony D James,[3,4]* & Xiao-Peng He[1,2]*

[1] *Key Laboratory for Advanced Materials and Joint International Research Laboratory of Precision Chemistry and Molecular Engineering, Feringa Nobel Prize Scientist Joint Research Center, School of Chemistry and Molecular Engineering, East China University of Science and Technology, 130 Meilong Rd., Shanghai 200237, China*
[2] *The International Cooperation Laboratory on Signal Transduction, Eastern Hepatobiliary Surgery Hospital, National Center for Liver Cancer, Shanghai 200438, China*
[3] *Department of Chemistry, University of Bath, Bath BA2 7AY, UK*
[4] *School of Chemistry and Chemical Engineering, Henan Normal University, Xinxiang 453007, China.*

*Corresponding authors.
xlhu@ecust.edu.cn (X.-L. Hu)
Hongyang_zhang@ecust.edu.cn (H.-Y. Zhang)
t.d.james@bath.ac.uk (T. D. James)
xphe@ecust.edu.cn (X.-P. He)

Contents list

S1. Experimental section
S2. Additional figures and tables
S3. Spectra of new compounds
S4. Additional references

S1. Experimental Section

Synthesis of compound 2. Compound **1** was synthesized according to a literature procedure.[1] To a stirred solution of pyridine (1.4 mL) was added $POCl_3$ (234 μL, 2.5 mmol) at 0 °C under a N_2 atmosphere. The resulting mixture was stirred for 5 min, and then **1** (200 mg in 2 mL tetrahydrofuran (THF), 0.65 mmol) was added dropwise at 0°C. The resulting mixture was stirred at 0 °C for 15 min, and then warmed to room temperature (r.t.) and stirred for another 3 h. Then, to the resulting mixture 3-hydroxypropionitrile (414 mg, 5.8 mmol) was added dropwise at 0 °C. The resulting mixture was stirred at r.t. for 16 h. The organic solvents were removed under reduced pressure, and the residue was diluted with 100 mL of 1 M $CuSO_4$ (aq.), and extracted with ethyl acetate. The combined organic layer was dried using Na_2SO_4, filtered, and concentrated under reduced pressure. The residue was purified by silica gel column chromatography eluted with MeOH/DCM (1/19) to afford compound **2** (260 mg, 81.2 %) as a yellow oil. ^{1}H NMR (400 MHz, $CDCl_3$) δ 7.94 (d, J = 16.0 Hz, 1H), 7.49 (d, J = 8.1 Hz, 1H), 7.20 (d, J = 8.2 Hz, 1H), 6.45 (d, J = 16.1 Hz, 1H), 4.55 – 4.39 (m, 4H), 3.80 (s, 3H), 3.52 (s, 3H), 2.99 – 2.91 (m, 2H), 2.59 – 2.47 (m, 2H), 2.06 – 1.92 (m, 2H); ^{13}C NMR (101 MHz, $CDCl_3$) δ 166.86, 145.48, 143.18, 138.16, 137.46, 128.36, 128.17, 126.99, 124.92, 124.90, 122.98, 121.08, 116.26, 77.43, 77.12, 76.80, 63.41, 63.36, 57.73, 52.04, 28.91, 28.60, 19.65, 19.57, 17.48; ^{31}P NMR (162 MHz, $CDCl_3$) δ -8.12.

Synthesis of CL-A1. To a stirred mixture of **2** (205 mg, 0.4 mmol) in MeOH (2 mL) was added NaOMe (2.1 mL, 1 mol L^{-1} in MeOH, 2.1 mmol) at 0 °C. The mixture was stirred at room temperature for 5 h, and then purified by reverse flash chromatography using the following conditions: column: C18 silica gel; mobile phase: MeOH in water – 5% to 50% gradient in 30 min; detector: UV 254 nm to afford **CL-A1** (120 mg, 77.4 %) as a yellow solid. ^{1}H NMR (400 MHz, D_2O) δ 7.95 (d, J = 16.0 Hz, 1H), 7.67 (d, J = 8.2 Hz, 1H), 7.21 (d, J = 8.1 Hz, 1H), 6.55 (dd, J = 16.1, 2.5 Hz, 1H), 3.53 (s, 3H), 2.91 (t, J = 8.0 Hz, 2H), 2.62 – 2.48 (m, 2H), 2.00 (p, J = 8.1 Hz, 2H); ^{13}C NMR (101 MHz, D_2O) δ 172.6, 147.8, 147.7, 142.6, 138.0, 135.9, 129.3, 127.3, 125.0, 124.5, 122.3, 57.3, 28.1, 27.6, 16.9; ^{31}P NMR (162 MHz, D2O) δ -4.72. HRMS (ESI, m/z): $[M - H]^-$ calcd for $C_{15}H_{15}ClO_7P$: 373.0249, found 373.0243.

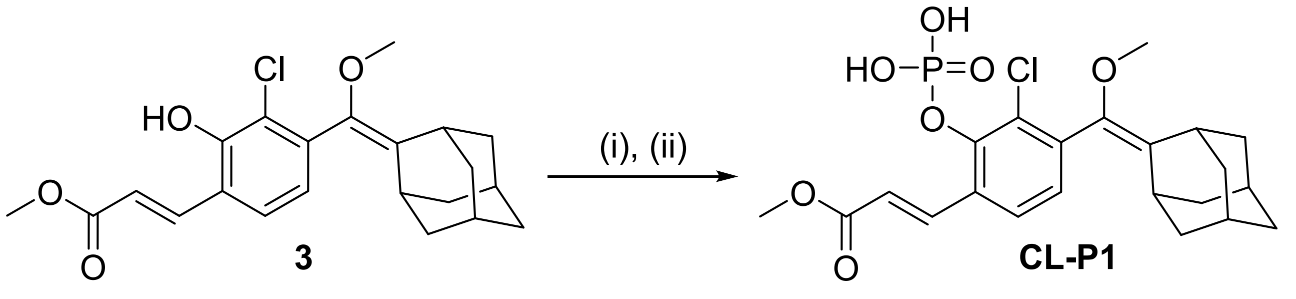

Scheme S1. Synthesis of **CL-P1**. Reagents and conditions: (i) $POCl_3$ in pyridine, 0 °C to r.t., 16 h; (ii) NaOMe in MeOH, r.t., 1 h.

Synthesis of CL-P1. Compound **3** was synthesized according to a literature procedure.[1] To a stirred solution of pyridine (140 μL) was added $POCl_3$ (19 μL, 0.20 mmol) at 0 °C under a N_2 atmosphere. The resulting mixture was stirred for 5 min, and then **3** (20 mg in 0.5 mL tetrahydrofuran (THF), 0.05 mmol) was added dropwise at 0 °C. The resulting mixture was stirred at 0 °C for 15 min, and then warmed to r.t. and stirred for another 3 h. Then, to the resulting mixture 3-hydroxypropionitrile (33 mg, 0.46 mmol) was added dropwise at 0 °C. The resulting mixture was stirred at r.t. for 16 h. The organic solvents were removed under reduced pressure, and the residue was diluted with 100 mL of 1 M $CuSO_4$ (aq.), and extracted with ethyl acetate. The combined organic layer was dried using Na_2SO_4, filtered, and concentrated under reduced pressure to afford a crude product. The crude product was used in the next step directly without further purification. The above crude product was dissolved in MeOH, and then NaOMe (250 μL, 1 mol L^{-1} in MeOH, 0.25 mmol) was added dropwise at r.t. The mixture was stirred at r.t. for 1 h, and then purified by reverse flash chromatography using the following conditions: column: C18 silica gel; mobile phase: MeOH in water 5% to 95% gradient in 30 min; detector: UV 254 nm to afford **CL-P1** (15 mg, 62.5 %) as a yellow solid. ^1H NMR (400 MHz, DMSO-d_6) δ 8.06 (d, J = 16.1 Hz, 1H), 7.83 (d, J = 8.1 Hz, 1H), 7.11 (d, J = 8.0 Hz, 1H), 6.66 (d, J = 16.1 Hz, 1H), 3.73 (s, 3H), 3.22 (s, 3H), 2.05 – 1.85 (m, 5H), 1.83 – 1.62 (m, 9H); ^{13}C NMR (101 MHz, DMSO-d_6) δ 166.90, 147.44, 139.98, 139.20, 137.59, 130.73, 129.06, 128.69, 127.99, 125.51, 120.28, 57.01, 52.04, 49.07, 40.61, 40.41, 40.20, 39.99, 39.78, 39.57, 39.36, 37.99, 36.93, 33.17, 32.79, 29.50, 29.20, 28.18, 28.01; ^{31}P NMR (162 MHz, DMSO-d_6) δ -6.21. HRMS (ESI, m/z): $[M - H]^-$ calcd for C22H25ClO7P: 467.1032, found 467.1024.

***In-situ* light-driven oxidation of CL-A1 and CL-P1.** To a white 96-well plate, 10 μL **CL-A1** (100 μM in deionized water) or **CL-P1**(1 mM in deionized water) and 10 μL **MB** (50 μM in deionized water) were sequentially added to 70 μL deionized water. After mixing with gentle pipetting, the mixture was irradiated under yellow light (590 nm, 10 W) for 30 min. After oxidation, the as-prepared solution was immediately used for enzymatic sensing.

Chemiluminescent kinetic measurements of CL-A1 and CL-P1. The stock solution of **CL-A1** and **CL-P1** was prepared in deionized water at a final concentration of 1 mM. Chemiluminescent kinetic profiles were recorded using a Synergy™ H4. The injector settings were fixed with a measuring time of 600 s and an interval time of 10 s. Measurements were conducted in a white 96-well plate. Each well contained 70 μL H_2O, 10 μL **CL-A1** (100 μM in water) or **CL-P1**(1 mM in deionized water), 10 μL **MB** (50 μM in water) and 10 μL ALP (1.6 U mL^{-1} in water) with a final well volume of 100 μL. H_2O, **MB** and **CL-A1** or **CL-P1** were added to the well in that order; after mixing with gentle pipetting, the mixture was irradiated under yellow light for 30 min, then ALP was introduced and the measurement was started immediately. Measurements were repeated three times to ensure reproducibility. For selectivity assay, a range of biospecies including α-glucosidase (α-Glc, 0.1 U mL^{-1}), β-galactosidase (β-Gal, 0.1 U mL^{-1}), glucose oxidase (GOD, 0.1 U mL^{-1}), human serum albumin (HSA, 50 mg mL^{-1}), Ca^{2+} (10 mM), Mg^{2+} (10 mM) and Zn^{2+} (10 mM) were incubated with the probe under identical conditions. CL responses were recorded on a microplate reader.

Chemiluminescent kinetic measurements of AMPPD and APS-5. The stock solution of **AMPPD** and **APS-5** stock solutions were prepared in deionized water at a final concentration of 1 mM. Chemiluminescent kinetic profiles were recorded using a Synergy™ H4. The injector settings were fixed with a measuring time of 600 s and an interval time of 10 s. Measurements were conducted in a white 96-well plate. Each well contained 70 μL Tris-HCl (50 mM, pH 9.0), 10 μL Sapphire-II, 10 μL ALP (0.16 U mL^{-1} in water) and 10 μL **AMPPD** or **APS-5** (1 mM in water) with a final well volume of 100 μL. ALP was added last during the assay. ALP was added at various concentrations to the well and the light emission was recorded immediately. Measurements were repeated three times to ensure reproducibility.

Bacterial strains and culture. *Escherichia coli* (*E. coli*) ATCC 25922 was obtained from ATCC (American Type Culture Collection), and other *E. coli* strains including DH5α, TOP10 and MG1655 were obtained from Beijing Tsingke Biotech Co., Ltd. Bacterial cells were first placed on Luria Bertani (LB) agar plates were transferred to LB culture medium, and then grown at 37 °C with shaking for 12 h.

Real-time quantitative polymerase chain reaction (RT-qPCR). Total RNA was isolated from cells using TRIzol Reagent (Accurate Biotechnology (human) Co., Ltd.) according to the manufacturer's protocol. Complementary DNA generated using a PrimeScript® RT reagent kit (ABclonal Biotech Co., Ltd) was analyzed by quantitative PCR using SYBR® Premix Ex TaqTM. RT-qPCR was performed using LightCycler ®Real-Time PCR System (F. Hoffmann La Roche). rsmD was detected as the housekeeping gene.

Primers for RT-qPCR were as follows.
rsmD-F forward, 5'- TGTTGACGCCCAATGTCTGG-3'
and reverse, 5'- TCTCAATCAACGTTGCCCCC-3'
phoA-MG1655 forward, 5′- TACCCCTGTGACAAAAGCCC-3'
and reverse, 5'- TGCGGCAGTAATTTCCGAGT-3'
phoA-ATCC25922-forward, 5'- GGGTGCAGTAATATCGCCCT-3'
and reverse, 5'- TTGCACTGGCACTCTTACCG-3'
phoA-DH5α-forward, 5'- GGGTGCAGTAATATCGCCCT-3'
and reverse, 5'- TTGCACTGGCACTCTTACCG-3'
phoA-Top10-forward, 5'- CCGGTAACGTTTCTACCGCA-3'
and reverse, 5'- ATTTTTCACTGGTCGCGCTC-3'

Melting curve analyses were carried out immediately after amplification to verify the specificity of the PCR amplification products. Fluorescence was measured at the end of the annealing extension phase of each cycle. A threshold value for the fluorescence of all samples was set manually. The reaction cycle at which the PCR product exceeds this fluorescence threshold was identified as the threshold cycle (CT). The relative mRNA expression level was determined by the $2^{-\Delta\Delta Ct}$ method.

Detection of bacterial ALP. Chemiluminescent kinetic profiles were recorded using a Synergy™ H4. The injector settings were fixed with a measuring time of 600 s and an interval time of 10 s. Measurements were conducted in a white 96-well plate. Each well contained 60 μL H_2O, 10 μL **CL-A1** (100 μM in water), 10 μL **MB** (50 μM in water) and 20 μL *E. coli* (1 × 10^9 CFU mL^{-1}) with a final well volume of 100 μL. H_2O, **MB** and **CL-A1** were added to the well in that order; after mixing with gentle pipetting, the mixture was irradiated under yellow light for 30 min, then *E. coli* was introduced and the measurement was started immediately. Measurements were repeated three times to ensure reproducibility. For the competition assay, (−)-*p*-bromolevamisole oxalate (***L-p*-BT**) oxalate was pretreated with ALP or *E. coli* for 30 min prior to addition of the probe.

Preparation of anti-AFP-magnetic beads. Magnetic beads (750 μL, 10 mg mL^{-1}) were added to a 1.5 mL centrifuge tube, followed by the addition of 200 μL EDCI (10 mg/mL in PBS (pH 7.4)) and 200 μL NHS (10 mg/mL in PBS (pH 7.4)). The resulting mixture was shaken at 37 °C for 30 min. Then, 0.23 mg of anti-AFP antibody dissolved in 70 μL of PBS (pH 7.4) was added to the system, followed by shaking at 37 °C for another 3 h. The resulting mixture was then magnetically separated and washed three times with 500 μL of wash buffer (TBST) and blocked with 1% BSA for 1 h at 37 °C to block non-specific binding sites. The magnetic beads were magnetically separated, washed again with wash buffer, and finally diluted with 1000 μL of deionized water.

Detection of AFP by CL immunoassays. The as-prepared anti-AFP antibody-modified magnetic beads (90 μL, 10 mg mL^{-1}) were added to a 1.5 mL centrifuge tube. Then, AFP antigen was added and incubate at 37 °C for 1 h. The resulting mixture was magnetically separated and washed three times with wash buffer (TBST). Then, rabbit anti-AFP (1:500) was added and incubated at 37 °C for 1 h. The resulting mixture was magnetically separated and washed with 50 μL goat antirabbit secondary antibody labeled with ALP (1:3000). After incubation at 37 °C for 1 h, the mixture was magnetically separated, wash again with wash buffer, and then diluted with 10 μL of deionized water. The magnetically separated species was added to a 70 μL serum-mimicking solution, followed by addition of 10 μL **CL-A1** (100 μM in deionized water) and 10 μL MB (50 μM in DMSO), sequentially. After mixing with gentle pipetting, the mixture was irradiated under yellow light for 30 min, and the generated emission intensity was detected on a Syn-ergy™ H4 microplate reader.

UHPLC-Q-TOF analysis. LC-MS data acquisition was conducted using a LC-MS hybrid system, which consisted of an ultra-high-performance liquid chromatography system (1290 Infinity, Agilent) coupled with a quadrupole time-of-flight mass analyzer (6530, Agilent). The Agilent Eclipse Plus C18 column (1.8 μm, 2.1 × 50 mm) was employed and maintained at a temperature of 65 °C. Water (containing 0.1% formic acid) was used as mobile phase A, acetonitrile as mobile phase B, and the flow rate was adjusted to 0.2 mL/min. The gradient profile (with respect to mobile phase B) was set as follows: 0.0–4.0 min, 30% → 100%. An electrospray ionization source (ESI) was utilized to collect data in negative ion mode. The source parameters were configured as follows: gas temperature 350 °C, drying gas 10 L/min, nebulizer 30 psi, capillary voltage 3000 V, and fragmentor voltage 150 V,

skimmer voltage 65 V, octopole RF voltage 750 V, mass scan range of m/z 50-1500. Two pairs of internal reference ions (m/z 121.0509, 922.0098 in positive mode and m/z 112.9856, 1033.9881 in negative mode) were chosen for the real-time mass correction during spectral acquisition. The targeted-MS/MS collision energy was selected as 20 or 40 eV depending on the difficulty of the precursor ion fragmentation.

S2. Additional figures and Tables

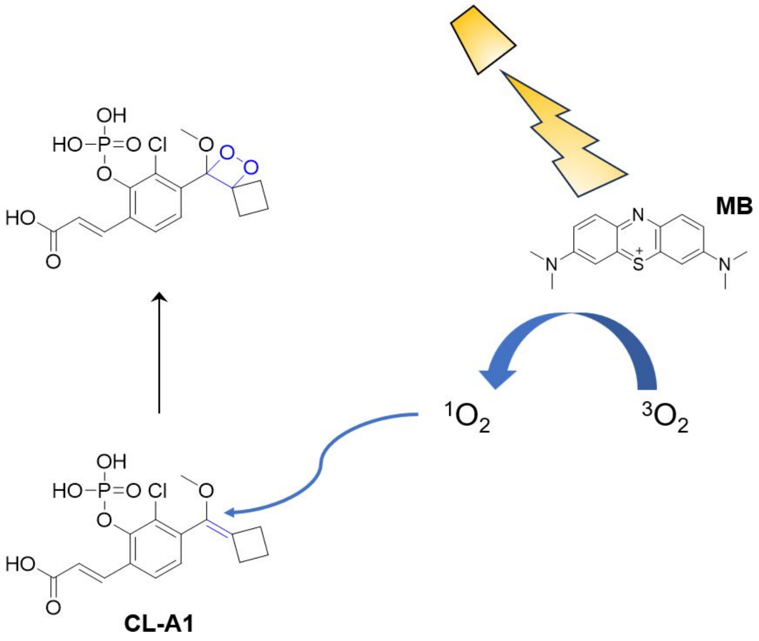

Figure S1. *In-situ* light-driven oxidation of **CL-A1**. MB is a known photosensitizer capable of producing $^1O_2$ under light irradiation. [2, 3] Then, the electron-rich enolether unit in **CL-A1** undergoes an oxidative reaction with $^1O_2$ to produce the active intermediate that can be hydrolyzed by ALP to produce chemiluminescence.

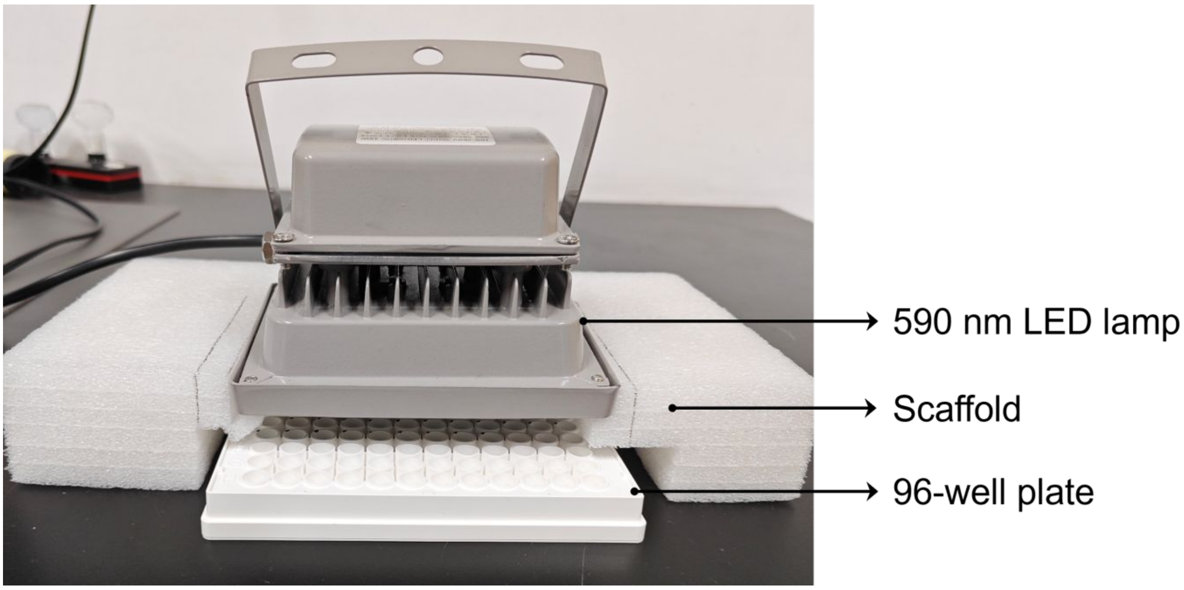

Figure S2. Experimental setup for the light-driven oxidation of **CL-A1**.

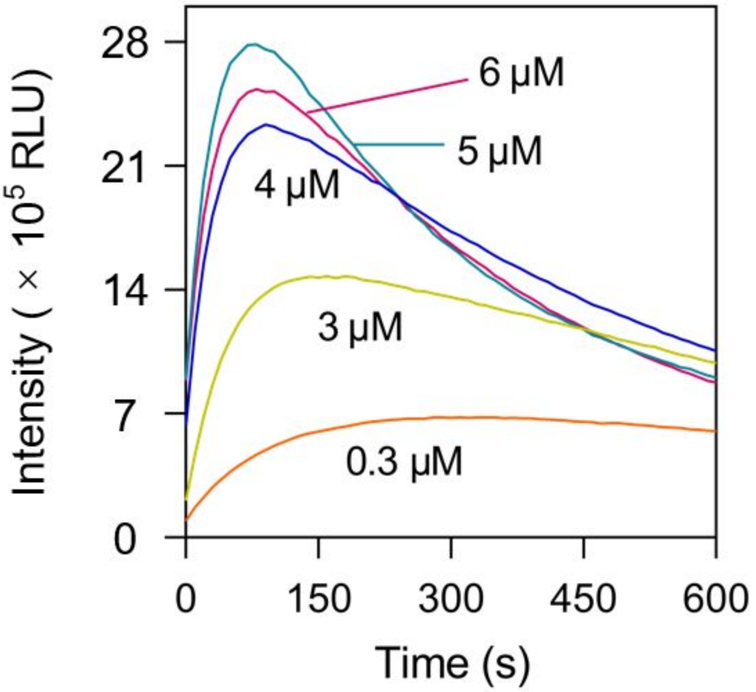

Figure S3. Time-dependent CL generation of **CL-A1** (10 μM) in the presence of ALP (0.16 U mL^{-1}) with different concentrations of **MB**. All measurements were performed in deionized water on a Synergy™ H4 microplate reader.

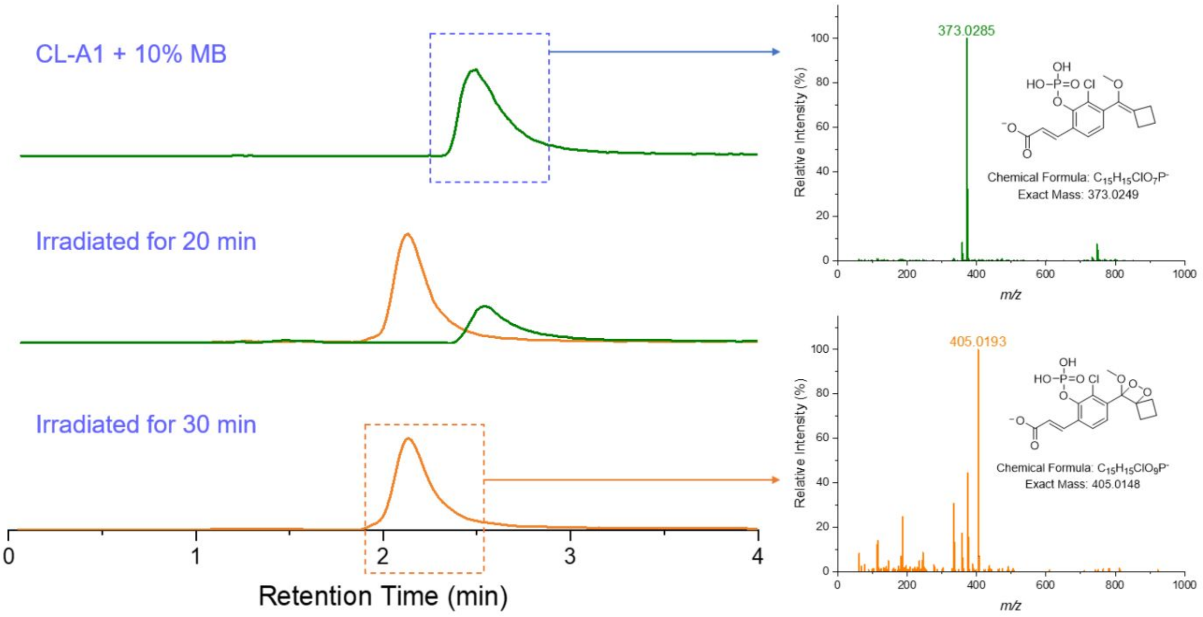

Figure S4. LC-MS analysis of the structural conversion of **CL-A1** to the active CL species after light irradiation (590 nm) for 20 min and 30 min.

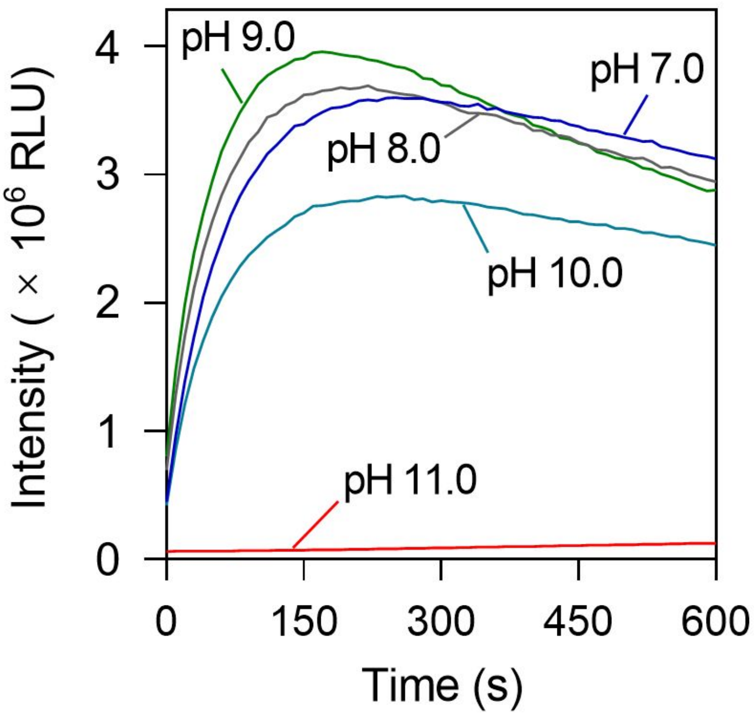

Figure S5. Time-dependent CL generation of **CL-A1** (10 μM) in the presence of ALP (0.16 U mL^{-1}) at different indicated pH. All measurements were performed in deionized water on a Syn-ergy™ H4 microplate reader.

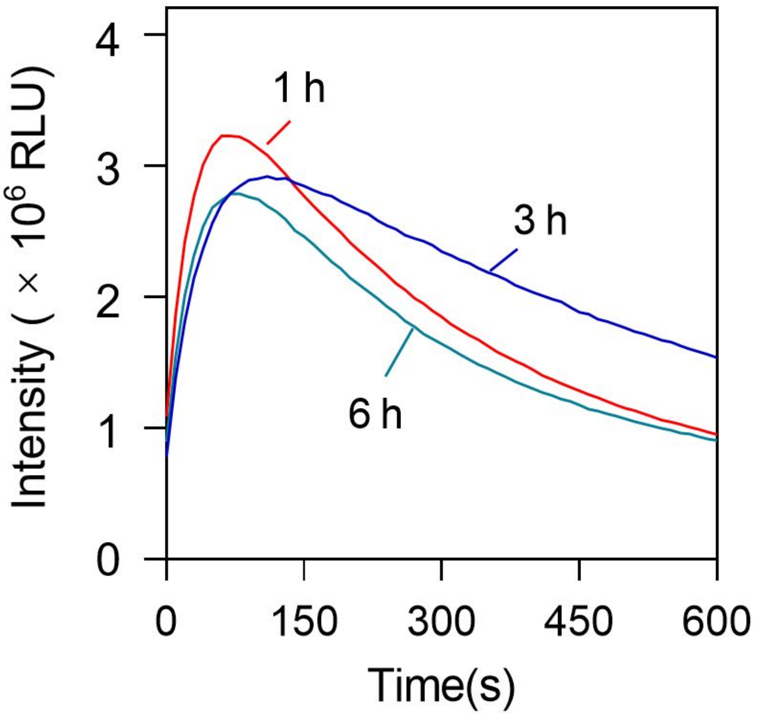

Figure S6. Time-dependent CL generation of **CL-A1** (10 μM) in the presence of ALP (0.16 U mL^{-1}). **CL-A1** was irradiated by white light for different times prior to analysis. All measurements were performed in deionized water on a Syn-ergy™ H4 microplate reader.

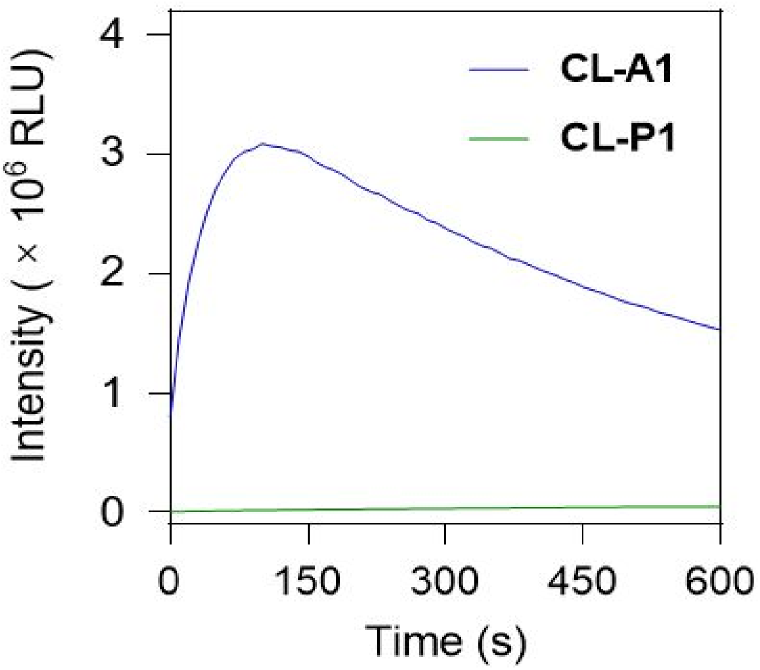

Figure S7. Time-dependent CL generation of **CL-A1** (10 μM) and **CL-P1** (100 μM) in the presence of ALP (0.16 U mL-1). All measurements were performed in deionized water on a Syn-ergy™ H4 microplate reader.

Table S1 Summary of the limit of detection (LOD) of reported probes for ALP

Probe name	LOD (U L^{-1})	Reference
**F-pY-LyP-1**	3.66	[4]
**P-CyPt**	0.098	[5]
**AMNF**	n.a.[1]	[6]
**TPEG-P**	0.059	[7]
**TPAPyP**	0.333	[8]
**P-CyFF-Gd**	0.0017	[9]
**DQM-ALP**	0.15	[10]
**Cl2–BDCM-ALP**	0.072	[11]
***f*-RCP**	0.061	[12]
**BOD-Py-PA**	n.a.[1]	[13]
**CL-A1**	**0.0075**	**This work**

[1]n.a. means not available.

Table S2 Recovery rate of AFP added to a serum-like solution determined by CL Immunoassay using **CL-A1** as the substrate

Sample (10^{-4} g mL^{-1})	Added (10^{-4} g mL^{-1})	Found (10^{-4} g mL^{-1})	Recovery rate (%)	RSD[a] (%, n = 3)
10	10	20.3	101.5	2.6
10	50	58.6	97.6	5.1
10	100	113.2	102.9	3.2

[a]RSD = Relative standard deviation.

S3. Spectra of new compounds

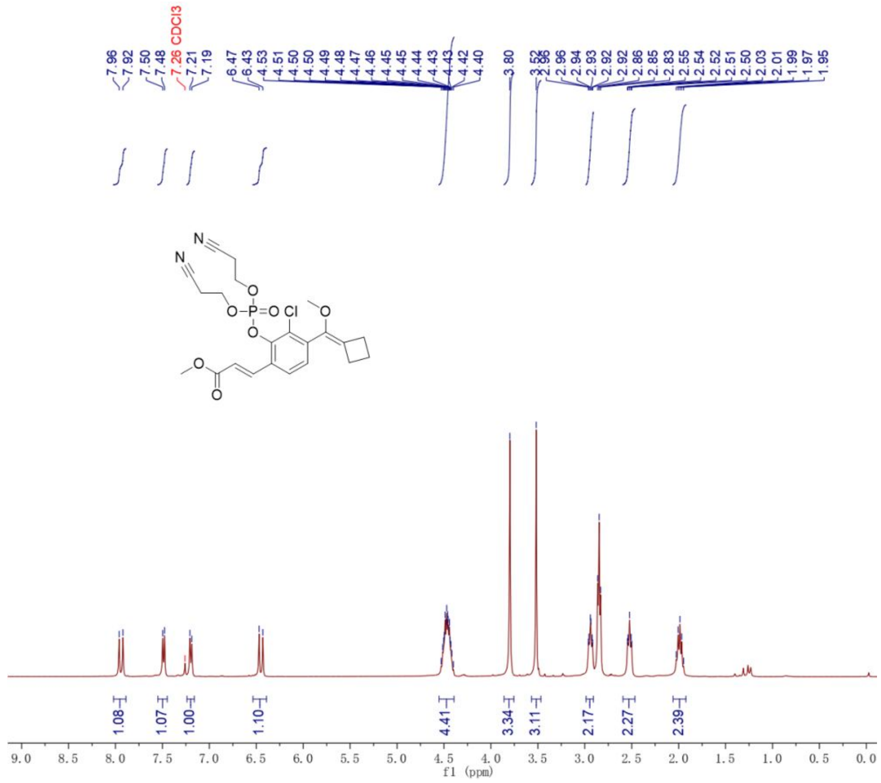

Figure S8. ^{1}H NMR spectrum of **compound 2**.

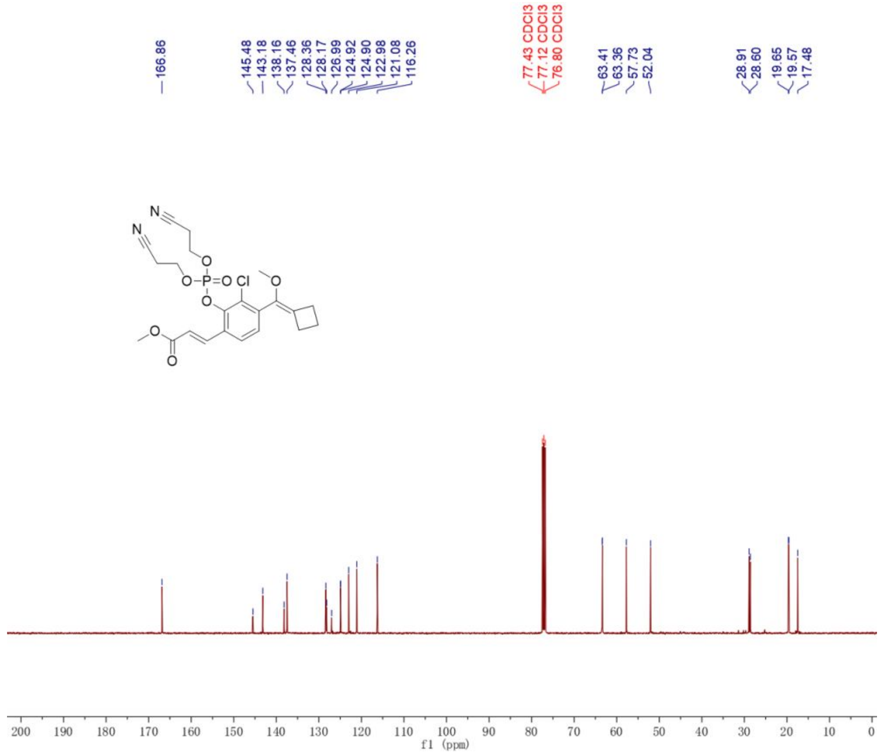

Figure S9. ^{13}C NMR spectrum of **compound 2**.

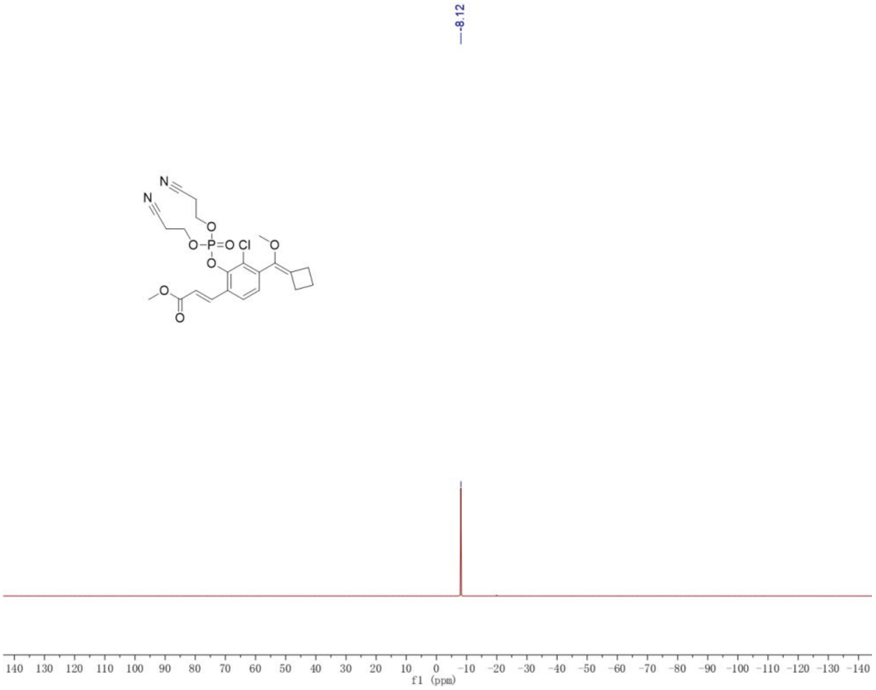

Figure S10. ^{31}P NMR spectrum of **compound 2**.

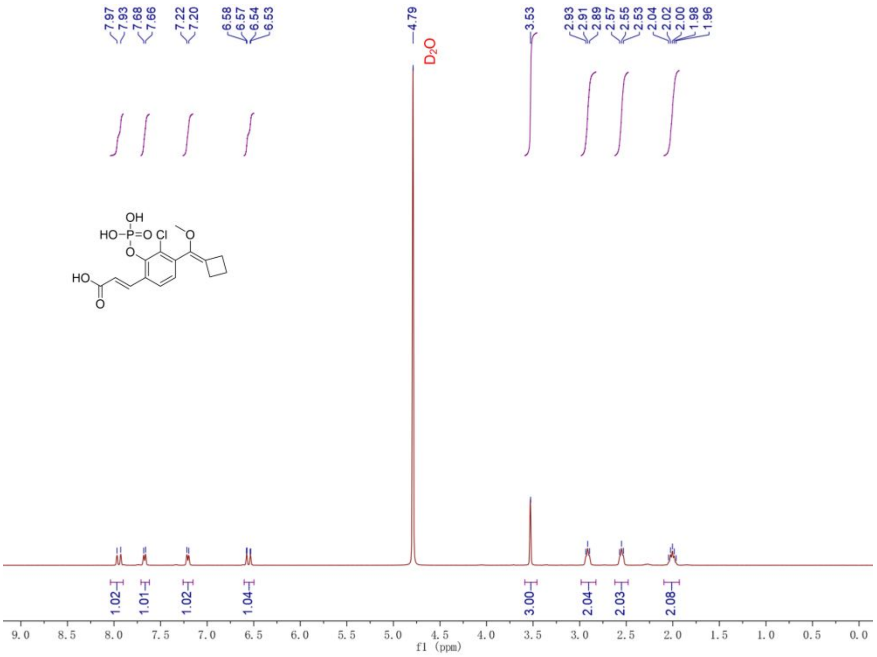

Figure S11. ^{1}H NMR spectrum of **CL-A1**.

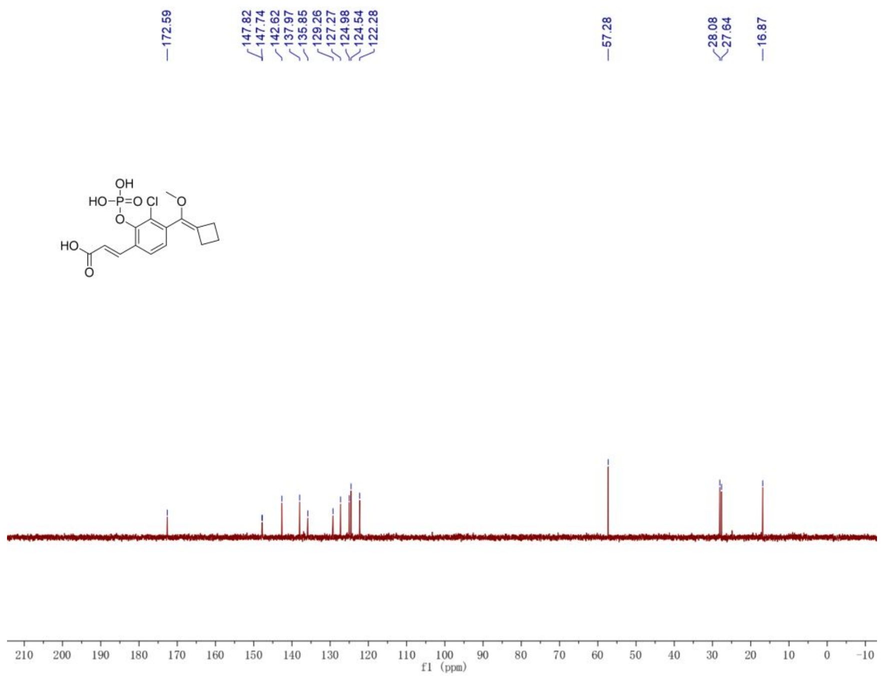

Figure S12. ^{13}C NMR spectrum of **CL-A1**.

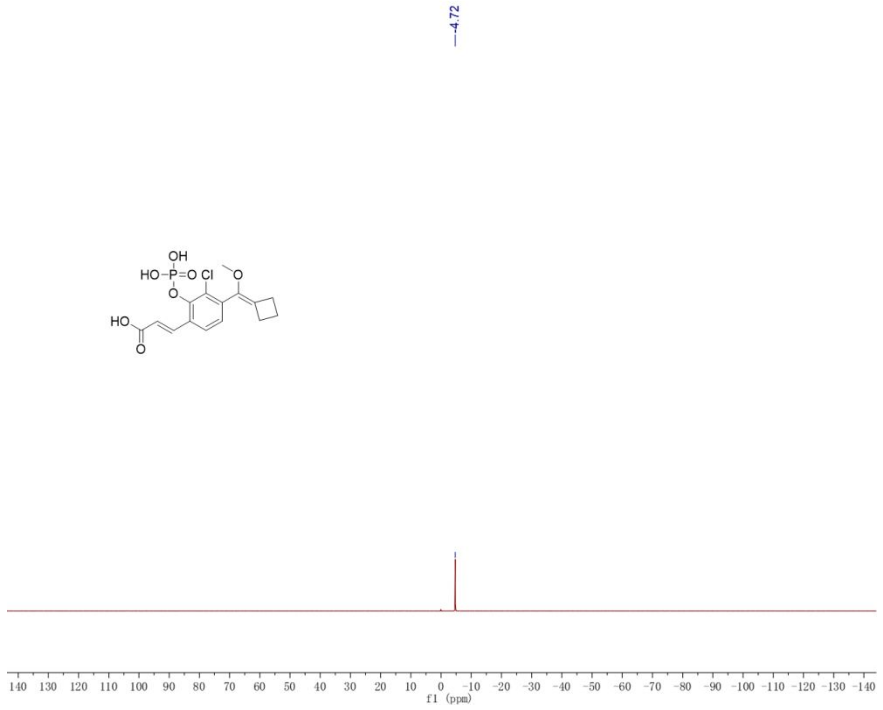

Figure S13. ^{31}P NMR spectrum of **CL-A1**.

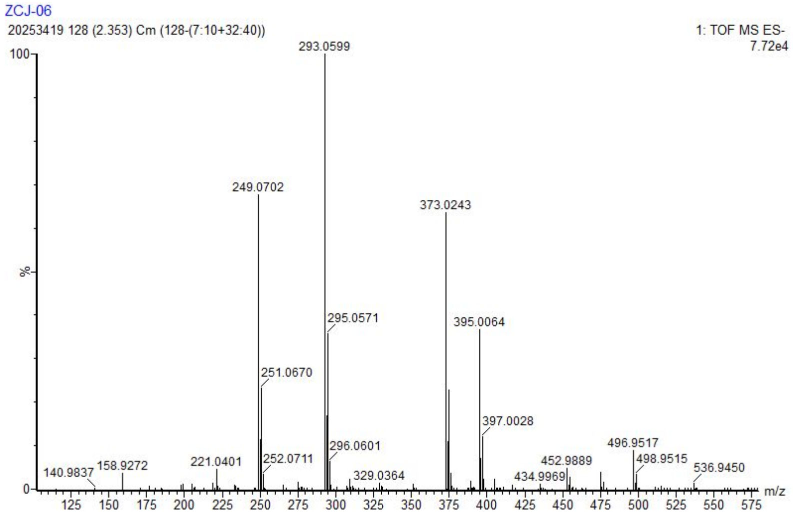

Figure S14. HRMS spectrum of **CL-A1**.

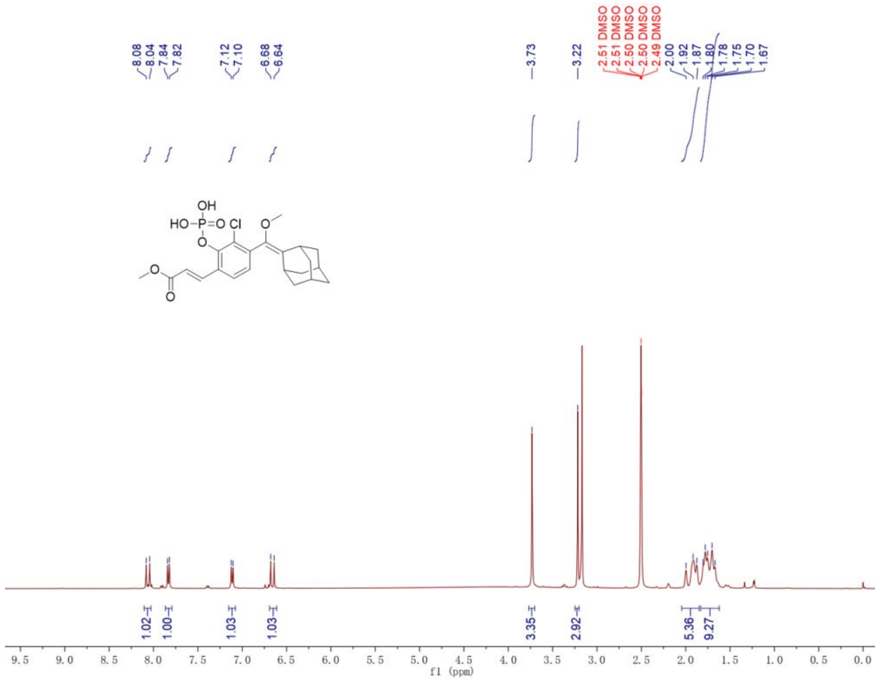

Figure S15. ^{1}H NMR spectrum of of **CL-P1**.

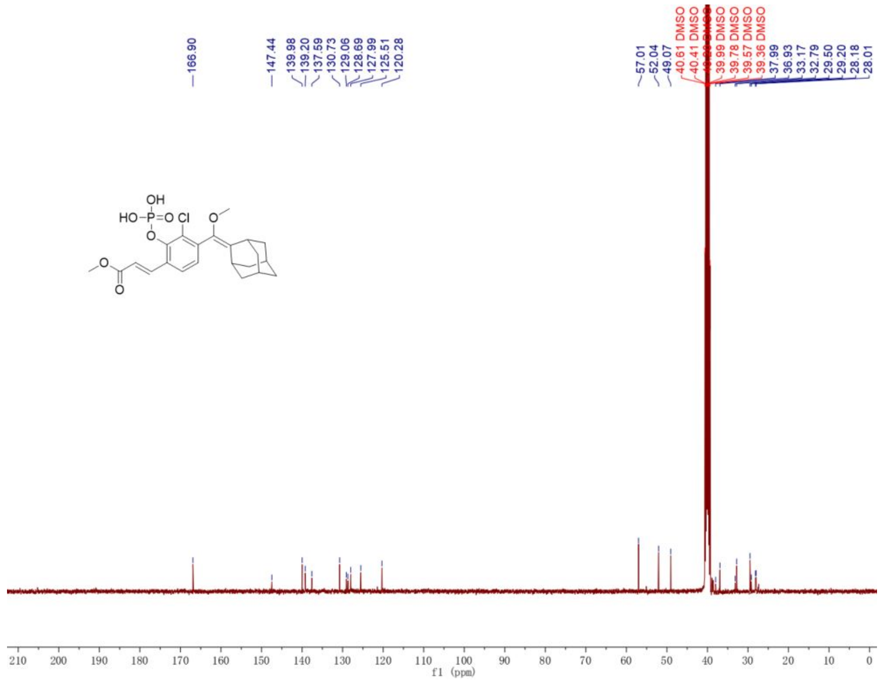

Figure S16. ^{13}C NMR spectrum of **CL-P1**.

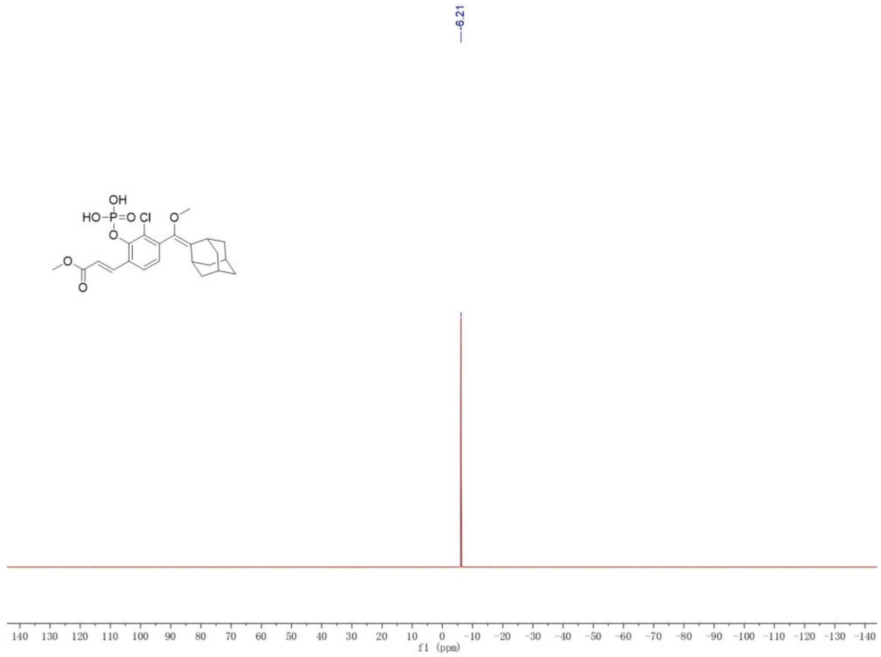

Figure S17. ^{31}P NMR spectrum of **CL-P1**.

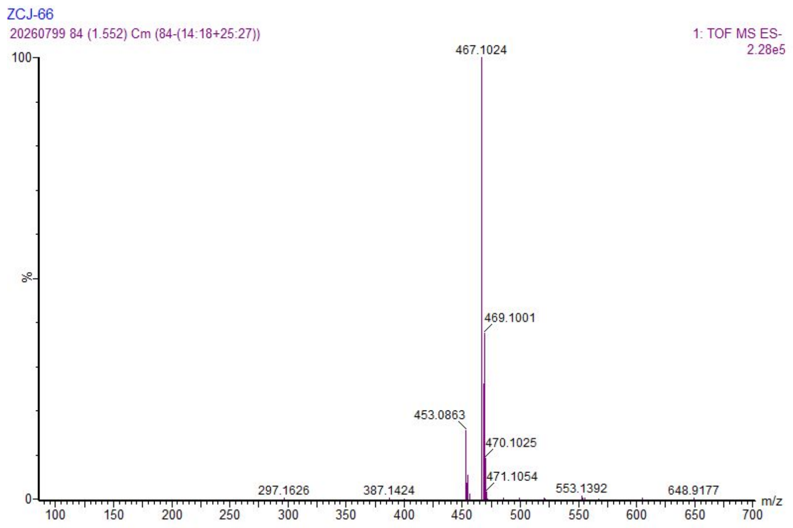

Figure S18. HRMS spectrum of **CL-P1**.

S4. Additional references

[1] Tannous, R.; Shelef, O.; Gutkin, S.; David, M.; Leirikh, T.; Ge, L.; Jaber, Q.; Zhou, Q.; Ma, P.; Fridman, M.; Spitz, U.; Houk, K. N.; Shabat, D. Spirostrain-accelerated Chemiexcitation of Dioxetanes Yields Unprecedented Detection Sensitivity in Chemiluminescence Bioassays. *ACS Central Sci.* **2024**, *10*, 28.

[2] Zhang, Y.; He, Y. -T.; He, X.; Huang, S.; Wang, X. -Q. Long-Term and Highly Sensitive Detection of Singlet Oxygen in Vivo with a Single Methylenecyclobutane Afterglow Probe. *CCS Chem*. **2025**, *7*, 3460.

[3] Xu, J. N.; Bonneviot, L.; Guari, Y.; Monnereau, C.; Zhang, K.; Poater, A.; Rodríguez-Pizarro, M.; Albela, B. Matrix Effect on Singlet Oxygen Generation Using Methylene Blue as Photosensitizer. *Inorganics*. **2024**, *12*(6), 155

[4] Li, B.; Wang, Y.; Chang, P.; Chen, H.; Zhu, Y.; Zhao, N.; Yang, Z.; Li, J. In Situ Enzyme Instructed Peptide Assembly Favoring a Three-target Sequentially Responsive Fluorescence Probe for the Early Identification of Atherosclerotic Plaque in Vivo. *Chem. Eng. J.* **2025**, *521*, 167162.

[5] Wen, X.; Zhang, R.; Hu, Y.; Wu, L.; Bai, H.; Song, D.; Wang, Y.; An, R.; Weng, J.; Zhang, S.; Wang, R.; Qiu, L.; Lin, J.; Gao, G.; Liu, H.; Guo, Z.; Ye, D. Controlled Sequential in Situ Self-assembly and Disassembly of a Fluorogenic Cisplatin Prodrug for Cancer Theranostics. *Nat. Commun.* **2023**, *14*, 800.

[6] Wang, Z.; Liang, H.; Liu, A.; Li, X.; Guan, L.; Li, L.; He, L.; Whittaker A. K.; Yang B.; Lin Q. Strength through unity: Alkaline Phosphatase-responsive AIEgen Nanoprobe for Aggregation-enhanced multi-mode Imaging and Photothermal Therapy of Metastatic Prostate Cancer. *Chin. Chem. Lett.* **2025**, *36*, 109765.

[7] Chen, X.; Shi, L.; Ran, X. -Y.; Zhang, L. -N.; Xie, K. -P.; Zhao, Y.; Chen, J.; Ye, L.; Yu, X. -Q.; Li, K. Rational Design of an Intramolecular Hydrogen Bond Enhanced Fluorescent Probe for Diagnosis of Drug-Induced Liver Injury. *ACS Mater. Lett.* **2024**, *6*,1059.

[8] Lam, K. W. K.; Chau, J. H. C.; Yu, E. Y.; Sun, F. Y.; Lam, J. W. Y.; Ding, D.; Kwok, R. T. K.; Sun, J.; He, X.; Tang, B. Z. An Alkaline Phosphatase-Responsive Aggregation-Induced Emission Photosensitizer for Selective Imaging and Photodynamic Therapy of Cancer Cells. *ACS nano* **2023**, *17*, 7145.

[9] Yan, R.; Hu, Y.; Liu, F.; Wei, S.; Fang, D.; Shuhendler, A. J.; Liu, H.; Chen, H.; Ye, D. Activatable NIR Fluorescence/MRI Bimodal Probes for in Vivo Imaging by Enzyme-Mediated Fluorogenic Reaction and Self-Assembly. *J. Am. Chem. Soc.* **2019**, *141*, 10331.

[10] Li, H.; Yao, Q.; Xu, F.; Li, Y.; Kim, D.; Chung, J.; Baek, G.; Wu, X.; Hillman, P. F.; Lee, E. Y.; Ge, H.; Fan, J.; Wang, J.; Nam, S.; Peng, X. -J.; Yoon, J. An Activatable AIEgen Probe for High-Fidelity Monitoring of Overexpressed Tumor Enzyme Activity and Its Application to Surgical Tumor Excision. *Angew. Chem. Int. Ed.* **2020**, *59*, 10272.

[11] Jie, X.; Wu, M.; Yang, H.; Wei, W. Red–Near-Infrared Fluorescent Probe for Time-Resolved in Vivo Alkaline Phosphatase Detection with the Assistance of a Photoresponsive Nanocontainer. *Anal. Chem.* **2019**, *91*, 13174.

[12] Fang, J.; Feng, Y.; Zhang, Y.; Wang, A.; Li, J.; Cui, C.; Guo, Y.; Zhu, J.; Lv, Z.; Zhao, Z.; Xu,

C.; Shi, H. Alkaline Phosphatase-Controllable and Red Light-Activated RNA Modification Approach for Precise Tumor Suppression. *J. Am. Chem. Soc.* **2022**, *144*, 23061.
[13] Wang, R.; Yin, K.; Ma, M.; Zhu, T.; Gao, J.; Sun, J.; Dong, X.; Dong, C.; Gu, X.; Tian, H.; Zhao, C. Alkaline Phosphatase-Initiated Sensitive Responsiveness of Activatable Probes to Hydrogen Sulfide for Accurate Cancer Imaging and Differentiation. *CCS Chem.* **2022**, *4*, 3715.
